# Effect of fatty liver disease on liver function and fibrosis in patients with chronic hepatitis B: a cross-sectional study

**DOI:** 10.3389/fmed.2024.1481051

**Published:** 2024-11-21

**Authors:** Xiaohui Fang, Yuhang Yin, Haonan Zhao, Cai’e Wang, Hui Li, Yiyang Shang, Jiayu Li, Yue Gao, Nahum Méndez-Sánchez, Xingshun Qi

**Affiliations:** ^1^Department of Gastroenterology, The General Hospital of Northern Theater Command (Teaching Hospital of Shenyang Pharmaceutical University), Shenyang, China; ^2^Department of Life Sciences and Biopharmaceutics, Shenyang Pharmaceutical University, Shenyang, China; ^3^Postgraduate College, China Medical University, Shenyang, China; ^4^Department of Laboratory Medicine, The General Hospital of Northern Theater Command, Shenyang, China; ^5^Department of Ultrasound, The General Hospital of Northern Theater Command, Shenyang, China; ^6^Liver Research Unit, Medica Sur Clinic and Foundation, National Autonomous University of Mexico, Mexico City, Mexico

**Keywords:** fatty liver disease, chronic hepatitis B, fibrosis, effect, prevalence

## Abstract

**Purpose:**

Chronic hepatitis B (CHB) and fatty liver disease (FLD) are common chronic liver diseases, both of which can progress to advanced liver diseases with poor outcome. However, it remains controversial whether the presence of FLD aggravates the disease severity of CHB patients.

**Patients and methods:**

All consecutive outpatients who were diagnosed with CHB at our department between March 1, 2021 and September 30, 2023 were retrospectively screened. They were divided into FLD and non-FLD groups. Liver function parameters and non-invasive indicators of liver fibrosis, including liver stiffness measurement (LSM) value, fibrosis-4 index (FIB-4) score, and aspartate aminotransferase to platelet ratio index (APRI) score, were compared between the two groups. Subgroups analyses were performed in HBeAg-positive, HBeAb-positive, HBV DNA > 10 IU/mL, mild FLD, and moderate/severe FLD patients.

**Results:**

Overall, 201 CHB patients were included, of whom 76 (37.81%) had FLD. In the overall analyses, CHB patients with FLD had a significantly higher alanine aminotransferase (ALT) (47.04 ± 53.28 vs. 32.95 ± 35.10, *p* = 0.003) than those without FLD, but there was no significant difference in the LSM value (7.79 ± 5.16 vs. 8.19 ± 4.99, *p* = 0.508), FIB-4 score (1.13 ± 0.75 vs. 1.28 ± 0.99, *p* = 0.679), and APRI score (0.41 ± 0.46 vs. 0.36 ± 0.47, *p* = 0.535) between CHB patients with and without FLD. The above-mentioned statistical results in all subgroup analyses were nearly consistent with those in the overall analyses.

**Conclusion:**

FLD may intensify abnormal liver function reflected by increased ALT level in CHB patients, but not influence the progression of liver fibrosis.

## 1 Introduction

Chronic hepatitis B (CHB) is a chronic liver disease caused by long-term infection with hepatitis B virus (HBV), affecting an estimated 316 million people, which poses a significant global health challenge (1). In CHB patients, fibrosis can be secondary to persistent inflammation with subsequent scar formation (2). Approximately 20% of them will progress from fibrosis to cirrhosis and hepatocellular carcinoma (HCC), in spite of widespread use of HBV vaccines and effective antiviral therapy in recent years (3, 4).

Fatty liver disease (FLD), a condition characterized by excessive fat accumulation in the liver, is mainly divided into alcohol-related and metabolic dysfunction-associated fatty liver disease (MAFLD) (5, 6). MAFLD is closely related to overweight/obesity, type 2 diabetes, and metabolic dysregulation, and it is the prominent cause of FLD and becomes the most common cause of chronic liver diseases in some regions (7–9). According to the recent findings from a large National Health and Nutrition Examination Surveys (NHANES) study, the estimated prevalence of MAFLD among the American adults significantly increased from 22% to 36% during the past three decades (10). Lifestyle modifications are the only approved interventions for the treatment of FLD (11). Once MAFLD patients developed fibrosis, the risk of liver-related mortality would be significantly increased (12).

Generally, CHB and FLD are common chronic liver diseases, both of which can progress to advanced liver diseases with poor prognosis. The coexistence of FLD with CHB is also common, especially in Asia (13, 14). Recently, some studies have shown that concomitant FLD may exacerbate the progression of CHB with a higher incidence of liver fibrosis and abnormal liver function, increasing the risk of cirrhosis, HCC, and death (13, 15, 16). However, others suggested that FLD might be beneficial to the disease course of CHB by decreasing the levels of HBV DNA and HBsAg, and compromising the development of liver fibrosis (17, 18). Considering this controversy in this topic, a retrospective study has been performed to explore the impact of FLD on liver function and fibrosis in patients with CHB.

## 2 Methods

### 2.1 Study design

We retrospectively reviewed the medical records of 201 outpatients with CHB who were treated by one physician (XQ) at the Department of Gastroenterology of the General Hospital of Northern Theater Command between March 1, 2021 and September 30, 2023. This study has been approved by the Medical Ethical Committee of the General Hospital of Northern Theater Command with an approval number [NO. Y (2024) 082]. It was performed according to the 1975 Declaration of Helsinki. Patients’ written informed consents were waived by the Medical Ethical Committee of our hospital due to the retrospective nature of this study.

Exclusion criteria were as follows: (i) repeated visits of the same patient; (ii) patients diagnosed with liver cirrhosis; (iii) patients diagnosed with HCC; and (iv) absence of imaging examinations, such as ultrasound, computed tomography (CT), or magnetic resonance imaging (MRI).

### 2.2 Laboratory test

Total bilirubin [(TBIL, reference range: 0–21 μmol/L, reagent CH0101003), direct bilirubin (DBIL, reference range: 0–8 μmol/L, reagent CH0101004), alanine aminotransferase (ALT, reference range: 7–40 U/L, reagent AUZ2390), aspartate aminotransferase (AST, reference range: 13–35 U/L, reagent AUZ2197), alkaline phosphatase (AKP, reference range: 50–135 U/L, reagent AUZ1959), gamma-glutamyl transferase (GGT, reference range: 7–45 U/L, reagent GS9051G), total bile acid (TBA, reference range: 0–10 μmol/L, reagent CH0101005)] were analyzed with reagents from Mike Laboratories (Mike, Sichuan, China) on a AUS800 automatic biochemical analyzer (Beckman Coulter, Suzhou, China); platelet count [PLT, reference range: 125–350 (×10^9^/L), reagent DS] were analyzed with reagents from Mindray Laboratories (Mindray, Shenzhen, China) on a BC-6800PLUS instrument (Mindray, Shenzhen, China); and alpha-fetoprotein (AFP, reference range: 0–7 ng/mL, reagent 105-002524-00) were analyzed with reagents from Mindray Laboratories (Mindray, Shenzhen, China) on a CL-6000i instrument (Mindray, Shenzhen, China) at the Department of Laboratory Medicine. Virological indicators [hepatitis B surface antigen (HBsAg, reagent IM4403001), hepatitis B surface antibody (HBsAb, reagent IM4403002), hepatitis B e antigen (HBeAg, reagent IM4403003), hepatitis B e antibody (HBeAb, reagent IM4403004), hepatitis B core antibody (HBcAb, reagent IM4403005)] were analyzed with reagents from Mike Laboratories (Mike, Sichuan, China) on a i3000B instrument (Mike, Sichuan, China) at the Department of Laboratory Medicine. Hepatitis B virus deoxyribonucleic acid (HBV DNA, reagent 20230403B) was analyzed with reagents from Northeast Pharmaceutical (Northeast Pharmaceutical, Shanghai, China) on a Gentier 9EB instrument (Northeast Pharmaceutical, Shanghai, China) by real-time fluorescence quantitative polymerase chain reaction at the Department of Laboratory Medicine.

### 2.3 Imaging

Hepatobiliary imaging examination (ultrasound, CT, or MRI) was performed at the Department of Ultrasound and Department of Radiology, when the patients should be fasting. The liver stiffness measurement (LSM) and controlled attenuation parameter (CAP) value were measured by the Hepatus 6 CS liver ultrasound diagnostic instrument (Mindray, Shenzhen, China) at the Department of Gastroenterology, when the patients should be fasting for at least 2 h (19).

### 2.4 Scores

Body mass index (BMI), fibrosis-4 index for liver fibrosis (FIB-4), and aspartate aminotransferase to platelet ratio index (APRI) were also calculated.

$\mathrm{BMI = weight (kg)}/{\mathrm{height}}^{2}\;\left(m^{2}\right)$

$\begin{array}{l} \mathrm{FIB}-4\;\mathrm{score}=\mathrm{age}\;\left(\mathrm{yr}\right)\times AST\;\left(U/L\right)/\\ \left[PLT\;\left(\times 10^{9}/L\right)\times {\mathrm{ALT}}^{1/2}\;\left(U/L\right)\right] \end{array}$

$\begin{array}{l} \mathrm{APRI score}=[AST\;\left(U/L\right)/\mathrm{upper limit of normal}\;\left(ULN\right)\;\\ \;\mathrm{of}\;AST\;\left(U/L\right)\times 100]/PLT\;\left(\times 10^{9}/L\right) \end{array}$

### 2.5 Diagnosis and group

CHB was diagnosed with positive HBsAg for a duration of more than 6 months, and antiviral therapy is recommended when HBV DNA level is positive (i.e., HBV DNA level is more than 10 IU/mL at our hospital) in CHB patients without cirrhosis according to the recommendations of current Chinese guideline on the prevention and treatment of chronic hepatitis B (20). Notably, HBV DNA screening should be further conducted when HBsAg is positive.

FLD was diagnosed under hepatobiliary ultrasound, CT, and/or MRI according to the recommendations of Chinese guideline on diagnosis and treatment for FLD (5). FLD was classified as mild (240–265 db/m), moderate (265–295 db/m), and severe (above 295 db/m) according to the CAP value. CHB patients were divided into FLD and non-FLD groups.

### 2.6 Statistical analyses

All statistical analyses were performed by using SPSS version 25.0 statistical software (IBM Corp, Armonk, NY, USA). Continuous variables were expressed as mean ± standard deviation and median (range), and compared by the independent sample t-tests for normal distribution, and those without normal distribution by nonparametric Mann–Whitney U tests. The Chi-square test was used for categorical variables to analyze the difference between groups. Subgroups analyses were performed in HBeAg-positive patients, HBeAb-positive patients, patients with HBV DNA > 10 IU/mL, and patients with mild and moderate/severe FLD. A two-tailed *p* < 0.05 was considered statistically significant.

## 3 Results

### 3.1 Patient selection

**Figure 1:**
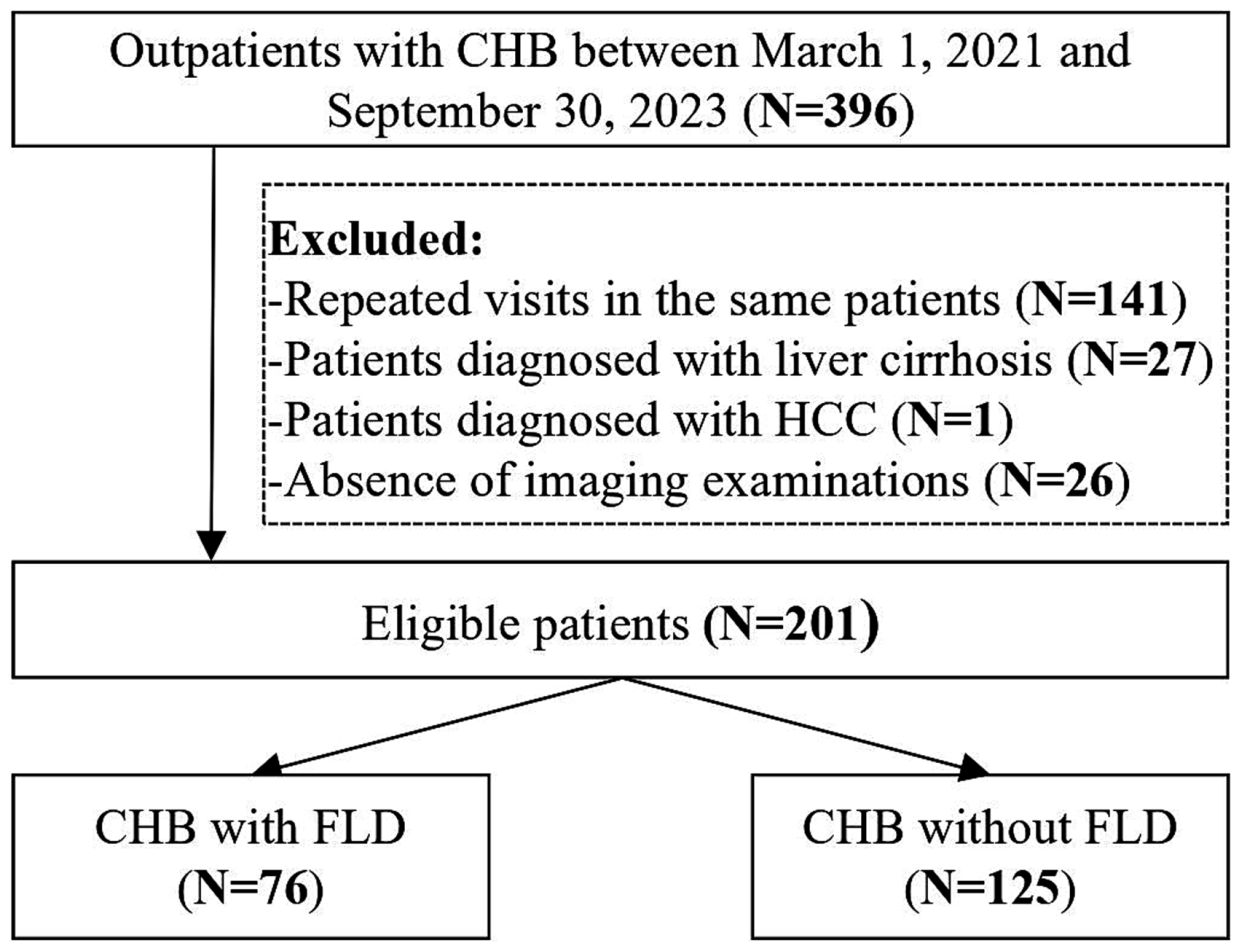
Flowchart of patients’ screening and grouping. CHB, chronic hepatitis B; FLD, fatty liver disease; HCC, hepatocellular carcinoma.

A total of 201 CHB patients were included (Figure 1), of whom 76 (37.81%) had FLD. Patient characteristics are summarized in Table 1.

**Table 1:** Baseline characteristics of CHB patients with versus without FLD.

Variables	CHB with FLD		CHB without FLD		*p* Value
	No. Pts	Median (range), Mean ± SD or Frequency (percentage)	No. Pts	Median (range), Mean ± SD or Frequency (percentage)	
Demographics					
Age (years)	76	42.00 (24.00–67.00); 44.14 ± 10.18	125	48.00 (18.00–79.00); 46.66 ± 12.83	0.126
Male (%)	76	43 (56.58%)	125	58 (46.40%)	0.162
BMI (kg/m^2^)	57	25.39 (15.24–59.88); 26.34 ± 6.04	97	22.89 (17.58–39.45); 23.45 ± 3.33	***0.001***
Laboratory parameters					
HBsAg >250 IU/mL (%)	76	61 (80.26%)	125	92 (73.60%)	0.283
HBsAg positive (%)	76	76 (100.00%)	125	125 (100.00%)	1.000
HBsAb positive (%)	75	3 (4.00%)	122	2 (1.64%)	0.578
HBeAg positive (%)	75	21 (28.00%)	122	37 (30.33%)	0.728
HBeAb positive (%)	75	52 (69.33%)	122	74 (60.66%)	0.218
HBcAb positive (%)	75	75 (100.00%)	122	118 (96.72%)	0.287
HBV DNA >10 IU/mL (%)	72	41 (56.94%)	122	68 (55.74%)	0.870
TBIL (μmol/L)	74	13.00 (3.60–38.30); 13.27 ± 5.80	122	11.85 (3.80–59.20); 13.20 ± 6.89	0.593
DBIL (μmol/L)	74	2.90 (1.20–19.66); 3.47 ± 2.55	122	2.95 (1.30–29.90); 3.71 ± 2.98	0.338
ALT (U/L)	74	28.69 (6.61–319.26); 47.04 ± 53.28	122	21.88 (6.44–260.50); 32.95 ± 35.10	***0.003***
AST (U/L)	74	24.66 (12.17–160.62); 33.39 ± 26.70	122	22.58 (13.08–155.58); 29.75 ± 22.30	0.091
AKP (U/L)	74	76.85 (18.81–120.41); 77.02 ± 20.06	122	77.23 (41.75–213.33); 79.49 ± 25.82	0.482
GGT (U/L)	74	24.77 (10.36–294.95); 40.06 ± 46.31	122	18.38 (7.74–430.38); 28.65 ± 44.90	***0.000***
TBA (μmol/L)	74	5.30 (0.40–22.30); 5.79 ± 3.77	122	5.70 (1.50–176.00); 8.30 ± 15.97	0.065
AFP (ng/mL)	67	2.40 (0.91–9.47); 2.75 ± 1.49	107	2.19 (0.73–274.45); 8.16 ± 33.70	0.741
**LSM (KPa)**	48	6.25 (3.90–35.80); 7.79 ± 5.16	72	6.45 (3.30–28.30); 8.19 ± 4.99	0.508
**CAP (db/m)**	48	258.35 (240.30–360.00); 265.08 ± 25.16	72	211.30 (143.80–240.00); 208.71 ± 21.67	***0.000***
**FIB-4 score**	46	1.05 (0.32–5.10); 1.13 ± 0.75	78	1.02 (0.35–7.27); 1.28 ± 0.99	0.679
**APRI score**	46	0.26 (0.12–2.21); 0.41 ± 0.46	78	0.24 (0.10–3.52); 0.36 ± 0.47	0.535

No. Pts, number of patients; CHB, chronic hepatitis B; BMI, body mass index; HBsAg, hepatitis B surface antigen; HBsAb, hepatitis B surface antibody; HBV DNA, hepatitis B virus deoxyribonucleic acid; HBeAg, hepatitis B e antigen; HBeAb, hepatitis B e antibody; HBcAb, hepatitis B core antibody; TBIL, total bilirubin; DBIL, direct bilirubin; ALT, alanine aminotransferase; AST, aspartate aminotransferase; AKP, alkaline phosphatase; GGT, gamma-glutamyl transferase; TBA, total bile acid; AFP, alpha-fetoprotein; LSM, liver stiffness measurement; CAP, controlled attenuation parameter; FIB-4, gibrosis-4 index; FLD, fatty liver disease; APRI, aspartate aminotransferase to platelet ratio index. Bold values mean there were significant differences between the two groups.

### 3.2 Overall analysis

CHB patients with FLD had significantly higher BMI (26.34 ± 6.04 vs. 23.45 ± 3.33, *p* = 0.001), ALT (47.04 ± 53.28 vs. 32.95 ± 35.10, *p* = 0.003), and GGT (40.06 ± 46.31 vs. 28.65 ± 44.90, *p* = 0.000) than those without FLD, but there was no significant difference in the proportions of HBsAg-positive >250 IU/mL (80.26% vs. 73.60%, *p* = 0.283), HBeAg-positive (28.00% vs. 30.33%, *p* = 0.728), HBeAb-positive (69.33% vs. 60.66%, *p* = 0.218), HBcAb-positive (100.00% vs. 96.72%, *p* = 0.287), and HBV DNA > 10 IU/mL (56.94% vs. 55.74%, *p* = 0.870), AST (33.39 ± 26.70 vs. 29.75 ± 22.30, *p* = 0.091), AFP (2.75 ± 1.49 vs. 8.16 ± 33.70, *p* = 0.741), LSM value (7.79 ± 5.16 vs. 8.19 ± 4.99, *p* = 0.508), FIB-4 score (1.13 ± 0.75 vs. 1.28 ± 0.99, *p* = 0.679), and APRI score (0.41 ± 0.46 vs. 0.36 ± 0.47, *p* = 0.535) between CHB patients with and without FLD (Table 1).

### 3.3 Subgroup analyses

#### 3.3.1 HBeAg-positive patients

In HBeAg-positive patients, the proportion of male (80.95% vs. 45.95%, *p* = 0.005) was significantly higher in CHB with FLD than those without FLD, but age (37.43 ± 7.41 vs. 41.19 ± 12.35, *p* = 0.153), BMI (24.49 ± 3.25 vs. 22.83 ± 3.32, *p* = 0.108), ALT (79.03 ± 85.33 vs. 53.61 ± 50.29, *p* = 0.394), AST (45.70 ± 41.28 vs. 43.71 ± 33.51, *p* = 0.973), GGT (34.44 ± 21.16 vs. 40.63 ± 68.66, *p* = 0.170), AFP (2.48 ± 1.29 vs. 21.51 ± 62.19, *p* = 0.135), LSM value (10.01 ± 7.61 vs. 9.52 ± 5.81, *p* = 0.899), FIB-4 score (0.86 ± 0.41 vs. 1.54 ± 1.57, *p* = 0.223), and APRI score (0.61 ± 0.73 vs. 0.62 ± 0.84, *p* = 0.530) were not significantly different between them (Table 2).

**Table 2:** Subgroups analyses of HBeAg-positive patients with versus without FLD.

Variables	CHB with FLD		CHB without FLD		*p* Value
	No. Pts	Median (range), Mean ± SD or Frequency (percentage)	No. Pts	Median (range), Mean ± SD or Frequency (percentage)	
Demographics					
Age (years)	21	38.00 (24.00–59.00); 37.43 ± 7.41	37	40.00 (18.00–71.00); 41.19 ± 12.35	0.153
Male (%)	21	17 (80.95%)	37	16 (45.95%)	***0.005***
BMI (kg/m^2^)	17	24.07 (18.42–30.86); 24.49 ± 3.25	28	22.79 (17.58–33.36); 22.83 ± 3.32	0.108
Laboratory parameters					
TBIL (μmol/L)	20	15.70 (6.30–25.20); 14.51 ± 5.42	37	11.60 (3.80–59.20); 14.05 ± 9.79	0.846
DBIL (μmol/L)	20	3.05 (1.60–5.80); 3.15 ± 1.23	37	3.10 (1.30–29.00); 4.52 ± 4.90	0.224
ALT (U/L)	20	45.03 (16.55–319.26); 79.03 ± 85.33	37	41.86 (9.13–260.50); 53.61 ± 50.29	0.394
AST (U/L)	20	27.94 (13.16–160.62); 45.70 ± 41.28	37	31.28 (16.23–155.58); 43.71 ± 33.51	0.973
AKP (U/L)	20	70.19 (44.52–116.53); 72.88 ± 18.20	37	88.13 (43.90–213.33); 87.59 ± 31.63	0.061
GGT (U/L)	20	32.14 (11.60–111.74); 34.44 ± 21.16	37	22.00 (7.74–430.38); 40.63 ± 68.66	0.170
TBA (μmol/L)	20	6.05 (0.40–22.30); 7.67 ± 5.36	37	7.10 (2.70–176.00); 13.27 ± 28.12	0.332
AFP (ng/mL)	17	2.12 (1.01–6.73); 2.48 ± 1.29	30	2.47 (1.27–274.45); 21.51 ± 62.19	0.135
**LSM (KPa)**	15	7.60 (4.60–35.80); 10.01 ± 7.61	21	7.30 (4.40–28.30); 9.52 ± 5.81	0.899
**CAP (db/m)**	15	250.80 (240.30–337.00); 259.88 ± 24.43	21	214.60 (165.10–240.00); 210.64 ± 20.76	***0.000***
**FIB-4 score**	9	0.72 (0.32–1.58); 0.86 ± 0.41	19	0.99 (0.45–7.27); 1.54 ± 1.57	0.223
**APRI score**	9	0.26 (0.12–2.21); 0.61 ± 0.73	19	0.36 (0.14–3.52); 0.62 ± 0.84	0.530

No. Pts, number of patients; CHB, chronic hepatitis B; BMI, body mass index; TBIL, total bilirubin; DBIL, direct bilirubin; ALT, alanine aminotransferase; AST, aspartate aminotransferase; AKP, alkaline phosphatase; GGT, gamma-glutamyl transferase; TBA, total bile acid; AFP, alpha-fetoprotein; LSM, liver stiffness measurement; CAP, controlled attenuation parameter; FIB-4, fibrosis-4 index; FLD, fatty liver disease; APRI, aspartate aminotransferase to platelet ratio index; HBeAg, hepatitis B e antigen. Bold values mean there were significant differences between the two groups.

#### 3.3.2 HBeAb-positive patients

In HBeAb-positive patients, BMI (27.12 ± 6.96 vs. 23.80 ± 3.58, *p* = 0.009), ALT (33.03 ± 22.87 vs. 24.35 ± 21.76, *p* = 0.004), AST (28.37 ± 16.91 vs. 23.91 ± 11.14, *p* = 0.039), and GGT (40.70 ± 53.26 vs. 23.25 ± 29.63, *p* = 0.002) were significantly higher in CHB patients with FLD than those without FLD, but the proportion of male (44.23% vs. 48.65%, *p* = 0.625), age (47.02 ± 10.04 vs. 48.82 ± 12.49, *p* = 0.553), AFP (2.81 ± 1.56 vs. 3.00 ± 3.35, *p* = 0.189), LSM value (6.78 ± 3.32 vs. 7.19 ± 4.25, *p* = 0.622), FIB-4 score (1.21 ± 0.81 vs. 1.21 ± 0.73, *p* = 0.996), and APRI score (0.36 ± 0.36 vs. 0.29 ± 0.21, *p* = 0.296) were not significantly different between them (Table 3).

**Table 3:** Subgroups analyses of HBeAb-positive patients with versus without FLD.

Variables	CHB with FLD		CHB without FLD		*p* Value
	No. Pts	Median (range), Mean ± SD or Frequency (percentage)	No. Pts	Median (range), Mean ± SD or Frequency (percentage)	
Demographics					
Age (years)	52	46.50 (25.00–67.00); 47.02 ± 10.04	74	48.00 (29.00–79.00); 48.82 ± 12.49	0.553
Male (%)	52	23 (44.23%)	74	36 (48.65%)	0.625
BMI (kg/m^2^)	38	26.69 (15.24–59.88); 27.12 ± 6.96	58	23.18 (19.03–39.45); 23.80 ± 3.58	***0.009***
Laboratory parameters					
TBIL (μmol/L)	51	11.70 (3.60–38.30); 12.90 ± 6.05	74	12.15 (3.80–31.20); 12.92 ± 5.16	0.987
DBIL (μmol/L)	51	2.80 (1.20–19.66); 3.63 ± 2.96	74	2.95 (1.30–8.90); 3.37 ± 1.41	0.427
ALT (U/L)	51	23.80 (6.61–115.85); 33.03 ± 22.87	74	19.16 (6.44–171.39); 24.35 ± 21.76	***0.004***
AST (U/L)	51	23.94 (12.17–108.90); 28.37 ± 16.91	74	21.72 (13.08–90.82); 23.91 ± 11.14	***0.039***
AKP (U/L)	51	78.03 (18.81–120.41); 78.05 ± 20.98	74	70.94 (41.75–161.37); 75.03 ± 23.12	0.457
GGT (U/L)	51	21.23 (10.36–294.95); 40.70 ± 53.26	74	16.64 (8.20–232.29); 23.25 ± 29.63	***0.002***
TBA (μmol/L)	51	5.00 (1.10–16.80); 5.10 ± 2.80	74	5.10 (1.50–19.00); 5.83 ± 3.29	0.325
AFP (ng/mL)	49	2.50 (0.91–9.47); 2.81 ± 1.56	68	2.06 (0.73–23.43); 3.00 ± 3.35	0.189
**LSM (KPa)**	31	5.70 (3.90–21.70); 6.78 ± 3.32	43	6.20 (3.30–25.40); 7.19 ± 4.25	0.622
**CAP (db/m)**	31	259.90 (243.90–360.00); 267.25 ± 25.91	43	208.00 (143.80–240.00); 204.91 ± 22.38	***0.000***
**FIB-4 score**	36	1.07 (0.39–5.10); 1.21 ± 0.81	53	1.05 (0.37–3.44); 1.21 ± 0.73	0.996
**APRI score**	36	0.27 (0.13–2.21); 0.36 ± 0.36	53	0.22 (0.10–1.38); 0.29 ± 0.21	0.296

No. Pts, number of patients; CHB, chronic hepatitis B; BMI, body mass index; TBIL, total bilirubin; DBIL, direct bilirubin; ALT, alanine aminotransferase; AST, aspartate aminotransferase; AKP, alkaline phosphatase; GGT, gamma-glutamyl transferase; TBA, total bile acid; AFP, alpha-fetoprotein; LSM, liver stiffness measurement; CAP, controlled attenuation parameter; FIB-4, fibrosis-4 index; FLD, fatty liver disease; APRI, aspartate aminotransferase to platelet ratio index; HBeAb, hepatitis B e antibody. Bold values mean there were significant differences between the two groups.

#### 3.3.3 HBV DNA > 10 IU/mL patients

In HBV DNA > 10 IU/mL patients, BMI (25.22 ± 3.54 vs. 23.10 ± 3.43, *p* = 0.009), ALT (62.47 ± 66.30 vs. 40.63 ± 43.99, *p* = 0.004), AST (41.02 ± 33.09 vs. 34.75 ± 28.33, *p* = 0.026), and GGT (38.13 ± 27.99 vs. 30.57 ± 51.87, *p* = 0.004) were significantly higher in CHB patients with FLD than those without FLD, but DBIL (3.04 ± 1.12 vs. 4.08 ± 3.73, *p* = 0.036) was significantly lower in CHB with FLD than those without FLD. The proportion of male (60.98% vs. 45.59%, *p* = 0.119), age (41.73 ± 9.77 vs. 45.51 ± 14.08, *p* = 0.283), AFP (2.63 ± 1.27 vs. 12.55 ± 46.20, *p* = 0.936), LSM value (8.74 ± 6.80 vs. 8.01 ± 5.05, *p* = 0.883), FIB-4 score (1.26 ± 0.98 vs. 1.34 ± 1.18, *p* = 0.995), and APRI score (0.56 ± 0.61 vs. 0.43 ± 0.61, *p* = 0.189) were not significantly different between them (Table 4).

**Table 4:** Subgroups analyses of HBV DNA > 10 IU/mL patients with versus without FLD.

Variables	CHB with FLD		CHB without FLD		*p* Value
	No. Pts	Median (range), Mean ± SD or Frequency (percentage)	No. Pts	Median (range), Mean ± SD or Frequency (percentage)	
Demographics					
Age (years)	41	40.00 (24.00–62.00); 41.73 ± 9.77	68	42.00 (18.00–79.00); 45.51 ± 14.08	0.283
Male (%)	41	25 (60.98%)	68	31 (45.59%)	0.119
BMI (kg/m^2^)	30	25.66 (18.42–31.35); 25.22 ± 3.54	52	22.89 (17.58–39.45); 23.10 ± 3.43	***0.009***
Laboratory parameters					
TBIL (μmol/L)	41	13.20 (5.60–25.20); 12.98 ± 4.81	67	12.00 (5.20–59.20); 13.97 ± 7.96	0.473
DBIL (μmol/L)	41	2.90 (1.60–5.80); 3.04 ± 1.12	67	3.10 (1.30–29.90); 4.08 ± 3.73	***0.036***
ALT (U/L)	41	37.66 (8.46–319.26); 62.47 ± 66.30	67	24.10 (9.13–260.50); 40.63 ± 43.99	***0.004***
AST (U/L)	41	26.64 (13.16–160.62); 41.02 ± 33.09	67	23.59 (14.59–155.58); 34.75 ± 28.33	***0.026***
AKP (U/L)	41	75.82 (44.52–117.54); 77.90 ± 19.29	67	72.62 (45.19–213.33); 79.54 ± 29.45	0.630
GGT (U/L)	41	31.67 (10.66–113.13); 38.13 ± 27.99	67	18.83 (9.56–430.38); 30.57 ± 51.87	***0.004***
TBA (μmol/L)	41	5.60 (0.40–22.30); 6.51 ± 4.21	67	5.90 (1.90–176.00); 9.66 ± 21.16	0.478
AFP (ng/mL)	36	2.29 (1.01–6.73); 2.63 ± 1.27	56	2.24 (0.73–274.45); 12.55 ± 46.20	0.936
**LSM (KPa)**	25	6.60 (3.90–35.80); 8.74 ± 6.80	38	6.65 (3.30–28.30); 8.01 ± 5.05	0.883
**CAP (db/m)**	25	253.20 (240.30–337.00); 261.57 ± 22.42	38	211.30 (143.80–240.00); 208.27 ± 22.92	***0.000***
**FIB-4 score**	22	1.06 (0.32–5.10); 1.26 ± 0.98	42	1.04 (0.35–7.27); 1.34 ± 1.18	0.955
**APRI score**	22	0.29 (0.12–2.21); 0.56 ± 0.61	42	0.26 (0.12–3.52); 0.43 ± 0.61	0.189

No. Pts, number of patients; CHB, chronic hepatitis B; BMI, body mass index; TBIL, total bilirubin; DBIL, direct bilirubin; ALT, alanine aminotransferase; AST, aspartate aminotransferase; AKP, alkaline phosphatase; GGT, gamma-glutamyl transferase; FLD, fatty liver disease; TBA, total bile acid; AFP, alpha-fetoprotein; LSM, liver stiffness measurement; CAP, controlled attenuation parameter; FIB-4, fibrosis-4 index; APRI, aspartate aminotransferase to platelet ratio index; HBV DNA, hepatitis B virus deoxyribonucleic acid. Bold values mean there were significant differences between the two groups.

#### 3.3.4 Mild FLD

In CHB patients, BMI (25.53 ± 3.78 vs. 23.45 ± 3.33, *p* = 0.005), and GGT (30.36 ± 25.07 vs. 28.65 ± 44.90, *p* = 0.049) were significantly higher in CHB patients with mild FLD than those without FLD, but the proportions of male (57.14% vs. 46.40%, *p* = 0.261), HBsAg-positive >250 IU/mL (80.00% vs. 73.60%, *p* = 0.440), HBeAg-positive (31.43% vs. 30.33%, *p* = 0.901), HBeAb-positive (65.71% vs. 60.66%, *p* = 0.587), HBcAb-positive (100.00% vs. 96.72%, *p* = 0.576), and HBV DNA > 10 IU/mL (54.55% vs. 55.74%, *p* = 0.903), age (44.60 ± 11.36 vs. 46.66 ± 12.83, *p* = 0.390), ALT (43.56 ± 52.54 vs. 32.95 ± 35.10, *p* = 0.101), AST (31.69 ± 26.24 vs. 29.75 ± 22.30, *p* = 0.229), AFP (2.90 ± 1.91 vs. 8.16 ± 33.70, *p* = 0.989), LSM value (8.27 ± 5.88 vs. 8.19 ± 4.99, *p* = 0.803), FIB-4 score (1.13 ± 0.43 vs. 1.28 ± 0.99, *p* = 0.854), and APRI score (0.40 ± 0.48 vs. 0.36 ± 0.47, *p* = 0.676) were not significantly different between them (Table 5).

**Table 5:** Subgroup analyses of CHB patients with mild FLD versus without FLD.

Variables	CHB with mild FLD		CHB without FLD		*p* Value
	No. Pts	Median (range), Mean ± SD or Frequency (percentage)	No. Pts	Median (range), Mean ± SD or Frequency (percentage)	
Demographics					
Age (years)	35	42.00 (24.00–67.00); 44.60 ± 11.36	125	48.00 (18.00–79.00); 46.66 ± 12.83	0.390
Male (%)	35	20 (57.14%)	125	58 (46.40%)	0.261
BMI (kg/m^2^)	29	24.93 (18.42–35.16); 25.53 ± 3.78	97	22.89 (17.58–39.45); 23.45 ± 3.33	***0.005***
Laboratory parameters					
HBsAg >250 IU/mL (%)	35	28 (80.00%)	125	92 (73.60%)	0.440
HBsAg positive (%)	35	35 (100.00%)	125	125 (100.00%)	1.000
HBsAb positive (%)	35	1 (2.86%)	122	2 (1.64%)	0.533
HBeAg positive (%)	35	11 (31.43%)	122	37 (30.33%)	0.901
HBeAb positive (%)	35	23 (65.71%)	122	74 (60.66%)	0.587
HBcAb positive (%)	35	35 (100.00%)	122	118 (96.72%)	0.576
HBV DNA >10 IU/mL (%)	33	18 (54.55%)	122	68 (55.74%)	0.903
TBIL (μmol/L)	33	11.30 (5.60–38.30); 13.03 ± 6.46	122	11.85 (3.80–59.20); 13.20 ± 6.89	0.908
DBIL (μmol/L)	33	2.80 (1.60–19.66); 3.75 ± 3.45	122	2.95 (1.30–29.90); 3.71 ± 2.98	0.346
ALT (U/L)	33	23.79 (11.43–269.30); 43.56 ± 52.54	122	21.88 (6.44–260.50); 32.95 ± 35.10	0.101
AST (U/L)	33	23.94 (13.16–160.62); 31.69 ± 26.24	122	22.58 (13.08–155.58); 29.75 ± 22.30	0.229
AKP (U/L)	33	74.34 (18.81–119.56); 75.13 ± 21.57	122	77.23 (41.75–213.33); 79.49 ± 25.82	0.375
GGT (U/L)	33	21.23 (11.60–111.74); 30.36 ± 25.07	122	18.38 (7.74–430.38); 28.65 ± 44.90	***0.049***
TBA (μmol/L)	33	5.00 (1.30–18.20); 5.64 ± 3.64	122	5.70 (1.50–176.00); 8.30 ± 15.97	0.085
AFP (ng/mL)	28	2.54 (0.91–9.47); 2.90 ± 1.91	107	2.19 (0.73–274.45); 8.16 ± 33.70	0.989
**LSM (KPa)**	35	6.60 (3.90–35.80); 8.27 ± 5.88	72	6.45 (3.30–28.30); 8.19 ± 4.99	0.803
**CAP (db/m)**	35	252.80 (240.30–264.60); 253.01 ± 6.90	72	211.30 (143.80–240.00); 208.71 ± 21.67	***0.000***
**FIB-4 score**	17	1.09 (0.45–2.14); 1.13 ± 0.43	78	1.02 (0.35–7.27); 1.28 ± 0.99	0.854
**APRI score**	17	0.29 (0.12–2.21); 0.40 ± 0.48	78	0.24 (0.10–3.52); 0.36 ± 0.47	0.676

No. Pts, number of patients; CHB, chronic hepatitis B; BMI, body mass index; HBsAg, hepatitis B surface antigen; HBsAb, hepatitis B surface antibody; HBV DNA, hepatitis B virus deoxyribonucleic acid; HBeAg, hepatitis B e antigen; HBeAb, hepatitis B e antibody; HBcAb, hepatitis B core antibody; FLD, fatty liver disease; TBIL, total bilirubin; DBIL, direct bilirubin; ALT, alanine aminotransferase; AST, aspartate aminotransferase; AKP, alkaline phosphatase; GGT, gamma-glutamyl transferase; TBA, total bile acid; AFP, alpha-fetoprotein; LSM, liver stiffness measurement; CAP, controlled attenuation parameter; FIB-4, fibrosis-4 index; APRI, aspartate aminotransferase to platelet ratio index. Bold values mean there were significant differences between the two groups.

#### 3.3.5 Moderate/severe FLD

In CHB patients, the proportions of male (53.85% vs. 46.40%, *p* = 0.609), HBsAg-positive >250 IU/mL (76.92% vs. 73.60%, *p* = 1.000), HBeAg-positive (30.77% vs. 30.33%, *p* = 1.000), HBeAb-positive (61.54% vs. 60.66%, *p* = 0.951), HBcAb-positive (100.00% vs. 96.72%, *p* = 1.000), and HBV DNA > 10 IU/mL (53.85% vs. 55.74%, *p* = 0.896), age (42.15 ± 11.16 vs. 46.66 ± 12.83, *p* = 0.225), ALT (37.06 ± 25.24 vs. 32.95 ± 35.10, *p* = 0.136), AST (25.81 ± 7.74 vs. 29.75 ± 22.30, *p* = 0.612), GGT (31.57 ± 20.28 vs. 28.65 ± 44.90, *p* = 0.200), AFP (2.47 ± 0.80 vs. 8.16 ± 33.70, *p* = 0.859), LSM value (6.50 ± 1.91 vs. 8.19 ± 4.99, *p* = 0.294), FIB-4 score (0.79 ± 0.41 vs. 1.28 ± 0.99, *p* = 0.066), and APRI score (0.23 ± 0.05 vs. 0.36 ± 0.47, *p* = 0.322) were not significantly different between them (Table 6).

**Table 6:** Subgroup analyses of CHB patients with moderate/severe FLD versus without FLD.

Variables	CHB with moderate/severe FLD		CHB without FLD		*p* Value
	No. Pts	Median (range), Mean ± SD or Frequency (percentage)	No. Pts	Median (range), Mean ± SD or Frequency (percentage)	
Demographics					
Age (years)	13	39.00 (31.00–64.00); 42.15 ± 11.16	125	48.00 (18.00–79.00); 46.66 ± 12.83	0.225
Male (%)	13	7 (53.85%)	125	58 (46.40%)	0.609
BMI (kg/m^2^)	12	26.54 (15.24–59.88); 29.08 ± 11.04	97	22.89 (17.58–39.45); 23.45 ± 3.33	0.106
Laboratory parameters					
HBsAg >250 IU/mL (%)	13	10 (76.92%)	125	92 (73.60%)	1.000
HBsAg positive (%)	13	13 (100.00%)	125	125 (100.00%)	1.000
HBsAb positive (%)	13	1 (7.69%)	122	2 (1.64%)	0.264
HBeAg positive (%)	13	4 (30.77%)	122	37 (30.33%)	1.000
HBeAb positive (%)	13	8 (61.54%)	122	74 (60.66%)	0.951
HBcAb positive (%)	13	13 (100.00%)	122	118 (96.72%)	1.000
HBV DNA >10 IU/mL (%)	13	7 (53.85%)	122	68 (55.74%)	0.896
TBIL (μmol/L)	13	13.90 (5.80–25.20); 15.32 ± 5.18	122	11.85 (3.80–59.20); 13.20 ± 6.89	0.082
DBIL (μmol/L)	13	3.10 (1.80–6.40); 3.33 ± 1.15	122	2.95 (1.30–29.90); 3.71 ± 2.98	0.797
ALT (U/L)	13	32.75 (8.46–101.68); 37.06 ± 25.24	122	21.88 (6.44–260.50); 32.95 ± 35.10	0.136
AST (U/L)	13	22.00 (19.02–43.72); 25.81 ± 7.74	122	22.58 (13.08–155.58); 29.75 ± 22.30	0.612
AKP (U/L)	13	68.00 (44.52–108.28); 70.05 ± 18.47	122	77.23 (41.75–213.33); 79.49 ± 25.82	0.202
GGT (U/L)	13	24.91 (10.36–68.10); 31.57 ± 20.28	122	18.38 (7.74–430.38); 28.65 ± 44.90	0.200
TBA (μmol/L)	13	5.10 (1.10–22.30); 5.66 ± 5.42	122	5.70 (1.50–176.00); 8.30 ± 15.97	0.091
AFP (ng/mL)	13	2.40 (1.28–4.35); 2.47 ± 0.80	107	2.19 (0.73–274.45); 8.16 ± 33.70	0.859
**LSM (KPa)**	13	6.20 (4.20–10.90); 6.50 ± 1.91	72	6.45 (3.30–28.30); 8.19 ± 4.99	0.294
**CAP (db/m)**	13	285.00 (269.00–360.00); 297.58 ± 27.83	72	211.30 (143.80–240.00); 208.71 ± 21.67	***0.000***
**FIB-4 score**	9	0.71 (0.32–1.56); 0.79 ± 0.41	78	1.02 (0.35–7.27); 1.28 ± 0.99	0.066
**APRI score**	9	0.22 (0.17–0.31); 0.23 ± 0.05	78	0.24 (0.10–3.52); 0.36 ± 0.47	0.322

No. Pts, number of patients; CHB, chronic hepatitis B; BMI, body mass index; HBsAg, hepatitis B surface antigen; HBsAb, hepatitis B surface antibody; HBV DNA, hepatitis B virus deoxyribonucleic acid; HBeAg, hepatitis B e antigen; HBeAb, hepatitis B e antibody; HBcAb, hepatitis B core antibody; FLD, fatty liver disease; TBIL, total bilirubin; DBIL, direct bilirubin; ALT, alanine aminotransferase; AST, aspartate aminotransferase; AKP, alkaline phosphatase; GGT, gamma-glutamyl transferase; TBA, total bile acid; AFP, alpha-fetoprotein; LSM, liver stiffness measurement; CAP, controlled attenuation parameter; FIB-4, fibrosis-4 index; APRI, aspartate aminotransferase to platelet ratio index. Bold values mean there were significant differences between the two groups.

## 4 Discussion

The first finding of our study should be that approximately 38% of CHB patients had FLD, which is close to the reported prevalence in three previous studies performed in China (36.5–41.8%), but a bit higher than the reported prevalence worldwide (34.9%) (13, 14, 21, 22). Despite so, a majority (35/48) of our patients who had measured CAP values had mild FLD, which is also consistent with previous studies (21, 22).

ALT, an enzyme mainly located in the cytoplasm of hepatocytes, is responsible for catalyzing the conversion of α-amino alanine to pyruvic acid (23). When liver tissue is damaged, ALT will leak into the systemic circulation from hepatocyte, causing an increase of ALT level in serum (23). Traditionally, ALT level is one of the important parameters for initiating antiviral therapy in HBsAg-positive patients (24, 25). It has been reported that increased ALT levels can be attributed to FLD in one fourth of the CHB patients (26). Our study also demonstrated that CHB patients with FLD had significantly higher ALT levels than those without, which was consistent with previous studies (15, 27, 28). Therefore, FLD might worsen liver damage in CHB patients.

Liver fibrosis, which refers to excessive accumulation of extracellular matrix proteins in the liver, is the consequence of chronic injury and inflammation of hepatocyte due to various pathogenic factors, such as HBV, hepatitis C virus, and other causes (29). As well known, liver biopsy is the gold standard for the assessment of liver fibrosis. However, it is often unacceptable due to its invasiveness and poor reproducibility (30). Thus, non-invasive methods for assessment of liver fibrosis have been frequently employed in clinical practice (31, 32). Several commonly used indicators have been recommended for the assessment of liver fibrosis by the European Association for the Study of the Liver (EASL) guidelines (33). Among them, LSM, FIB-4, and APRI are clinically significant markers for liver fibrosis among MAFLD patients (34–37). Our study demonstrated that the severity of liver fibrosis reflected by LSM, FIB-4, and APRI was not significantly influenced by the presence of FLD at both HBeAg-positive and HBeAb-positive stages, which was also supported by previous studies (38–41). This could be attributed to the fact that the majority of these patients from previous studies and ours had only mild FLD, which might hardly affect the development and progression of liver fibrosis (21, 22, 29).

The strength of our study is that all patients were treated by the same physician, potentially minimizing the heterogeneity in diagnosis and treatment selection among practitioners. However, our study also has some limitations. First, due to a relatively small sample size, the statistical results should be cautiously interpreted. Second, because our study population were outpatients and the nature of our study was retrospective, some information was not collected, such as history of alcohol abuse and metabolic variables (i.e., glucose, uric acid, and lipids). Thus, it was not possible to distinguish whether FLD was related to metabolic disorders in our patients. Third, due to the cross-sectional design of our study, the follow-up outcome was not evaluated.

## 5 Conclusion

FLD is common in CHB patients, and can intensify liver damage, particularly ALT level, but may not influence the progression of liver fibrosis. Large-scale cohort studies are imperative to further investigate the impact of FLD on virological markers and long-term outcome in CHB patients.

## Data Availability

The datasets presented in this article are not readily available because our data is not open to the public. Requests to access the datasets should be directed to Xingshun Qi, xingshunqi@126.com.

## References

[1] Collaborators GHB. Global, regional, and national burden of hepatitis B, 1990-2019: a systematic analysis for the global burden of disease study 2019. Lancet Gastroenterol Hepatol. (2022) 7:796–829. doi: 10.1016/S2468-1253(22)00124-8, PMID: 35738290 PMC9349325

[2] Bera C Hamdan-Perez N Patel K. Non-invasive assessment of liver fibrosis in hepatitis B patients. J Clin Med. (2024) 13:1046. doi: 10.3390/jcm13041046, PMID: 38398358 PMC10889471

[3] Ganem D Prince AM. Hepatitis B virus infection — natural history and clinical consequences. N Engl J Med. (2004) 350:1118–29. doi: 10.1056/NEJMra031087, PMID: 15014185

[4] Broquetas T Carrión JA. Past, present, and future of long-term treatment for hepatitis B virus. World J Gastroenterol. (2023) 29:3964–83. doi: 10.3748/wjg.v29.i25.3964, PMID: 37476586 PMC10354584

[5] Committee of Hepatology CRHA, Fatty Liver Expert Committee, Chinese Medical Doctor Association, National Workshop on Fatty Liver and Alcoholic Liver Disease, Chinese Society of Hepatology; National Workshop on Liver and Metabolism, Chinese Society of Endocrinology, Chinese Medical Association. Expert recommendations on standardized diagnosis and treatment for fatty liver disease in China (2019 revised edition). Zhonghua Gan Zang Bing Za Zhi. (2019) 27:748–53. doi: 10.3760/cma.j.issn.1007-3418.2019.10.003 31734987 PMC12769769

[6] Testino G Pellicano R. Corrected and republished from: metabolic associated liver disease. Panminerva Med. (2023) 65:391–9. doi: 10.23736/S0031-0808.23.04850-4, PMID: 37750860

[7] Wang C Kanaan G Shang Y Chai L Li H Qi X. Silymarin for treatment of adults with nonalcoholic fatty liver disease. Cochrane Database Syst Rev. (2023) 2023:CD015524. doi: 10.1002/14651858.CD015524 PMC12186484 40552569

[8] Eslam M Newsome PN Sarin SK Anstee QM Targher G Romero-Gomez M. A new definition for metabolic dysfunction-associated fatty liver disease: an international expert consensus statement. J Hepatol. (2020) 73:202–9. doi: 10.1016/j.jhep.2020.03.039, PMID: 32278004

[9] Méndez-Sánchez N Bugianesi E Gish RG Lammert F Tilg H Nguyen MH. Global multi-stakeholder endorsement of the MAFLD definition. Lancet Gastroenterol Hepatol. (2022) 7:388–90. doi: 10.1016/S2468-1253(22)00062-0, PMID: 35248211

[10] Xie ZQ Li HX Wang BK Yang ZM Zhang ZY Tan WL. Trends in prevalence and all-cause mortality of metabolic dysfunction-associated fatty liver disease among adults in the past three decades: results from the NHANES study. Eur J Intern Med. (2023) 110:62–70. doi: 10.1016/j.ejim.2023.01.029, PMID: 36754655

[11] Rinella ME Neuschwander-Tetri BA Siddiqui MS Abdelmalek MF Caldwell S Barb D. AASLD practice guidance on the clinical assessment and management of nonalcoholic fatty liver disease. Hepatology. (2023) 77:1797–835. doi: 10.1097/HEP.0000000000000323, PMID: 36727674 PMC10735173

[12] Ng CH Lim WH Hui Lim GE Hao Tan DJ Syn N Muthiah MD. Mortality outcomes by fibrosis stage in nonalcoholic fatty liver disease: a systematic review and Meta-analysis. Clin Gastroenterol Hepatol. (2023) 21:931–9.e5. doi: 10.1016/j.cgh.2022.04.014, PMID: 35513235 PMC10792524

[13] Zhou R Yang L Zhang B Gu Y Kong T Zhang W. Clinical impact of hepatic steatosis on chronic hepatitis B patients in Asia: a systematic review and meta-analysis. J Viral Hepat. (2023) 30:793–802. doi: 10.1111/jvh.13872, PMID: 37533208

[14] Jiang D Chen C Liu X Huang C Yan D Zhang X. Concurrence and impact of hepatic steatosis on chronic hepatitis B patients: a systematic review and meta-analysis. Ann Transl Med. (2021) 9:1718. doi: 10.21037/atm-21-3052, PMID: 35071412 PMC8743703

[15] Choi HSJ Brouwer WP Zanjir WMR de Man RA Feld JJ Hansen BE. Nonalcoholic steatohepatitis is associated with liver-related outcomes and all-cause mortality in chronic hepatitis B. Hepatology. (2020) 71:539–48. doi: 10.1002/hep.30857, PMID: 31309589

[16] Chan AW Wong GL Chan HY Tong JH Yu YH Choi PC. Concurrent fatty liver increases risk of hepatocellular carcinoma among patients with chronic hepatitis B. J Gastroenterol Hepatol. (2017) 32:667–76. doi: 10.1111/jgh.13536, PMID: 27547913

[17] Zhang M Chen S Wu X Zhou J Wang T Liu H. Persistent steatosis correlates with decreased fibrosis regression during anti-HBV treatment in patients with chronic HBV infection. J Med Virol. (2023) 95:e29156. doi: 10.1002/jmv.29156, PMID: 37822064

[18] Yi S Ren G Zhu Y Cong Q. Correlation analysis of hepatic steatosis and hepatitis B virus: a cross-sectional study. Virol J. (2024) 21:22. doi: 10.1186/s12985-023-02277-8, PMID: 38243304 PMC10799397

[19] Dietrich CF Bamber J Berzigotti A Bota S Cantisani V Castera L. EFSUMB guidelines and recommendations on the clinical use of liver ultrasound Elastography, update 2017 (long version). Ultraschall in der Medizin. (2017) 38:e48. doi: 10.1055/a-0641-0076 30176678

[20] Chinese Society of Hepatology CMA, Chinese Society of Infectious Diseases, Chinese Medical Association. Guidelines for the prevention and treatment of chronic hepatitis B (version 2022). Zhonghua Gan Zang Bing Za Zhi. (2022) 30:1309–31. doi: 10.3760/cma.j.cn501113-20221204-00607 36891718 PMC12677433

[21] Mi YQ Shi QY Xu L Shi RF Liu YG Li P. Controlled attenuation parameter for noninvasive assessment of hepatic steatosis using Fibroscan®: validation in chronic hepatitis B. Dig Dis Sci. (2015) 60:243–51. doi: 10.1007/s10620-014-3341-x, PMID: 25194851

[22] Kang NL Zhang JM Liu YR Lin S Dong J Jiang JJ. Novel predictive models using serum ceruloplasmin levels for hepatic steatosis in patients with chronic hepatitis B infection. Clin Res Hepatol Gastroenterol. (2020) 44:57–65. doi: 10.1016/j.clinre.2019.04.001, PMID: 31076363

[23] Nguyen LH Chao D Lim JK Ayoub W Nguyen MH. Histologic changes in liver tissue from patients with chronic hepatitis B and minimal increases in levels of alanine aminotransferase: a meta-analysis and systematic review. Clin Gastroenterol Hepatol. (2014) 12:1262–6. doi: 10.1016/j.cgh.2013.11.038, PMID: 24361419

[24] Terrault NA Lok ASF McMahon BJ Chang KM Hwang JP Jonas MM. Update on prevention, diagnosis, and treatment of chronic hepatitis B: AASLD 2018 hepatitis B guidance. Hepatology. (2018) 67:1560–99. doi: 10.1002/hep.29800, PMID: 29405329 PMC5975958

[25] Liver EAftSot. EASL 2017 clinical practice guidelines on the management of hepatitis B virus infection. J Hepatol. (2017) 67:370–98. doi: 10.1016/j.jhep.2017.03.021 28427875

[26] Spradling PR Bulkow L Teshale EH Negus S Homan C Simons B. Prevalence and causes of elevated serum aminotransferase levels in a population-based cohort of persons with chronic hepatitis B virus infection. J Hepatol. (2014) 61:785–91. doi: 10.1016/j.jhep.2014.05.045 24911461

[27] Prati D Taioli E Zanella A Della Torre E Butelli S Del Vecchio E. Updated definitions of healthy ranges for serum alanine aminotransferase levels. Ann Intern Med. (2002) 137:1–10. doi: 10.7326/0003-4819-137-1-200207020-00006, PMID: 12093239

[28] Hui RWH Seto WK Cheung KS Mak LY Liu KSH Fung J. Inverse relationship between hepatic steatosis and hepatitis B viremia: results of a large case-control study. J Viral Hepat. (2018) 25:97–104. doi: 10.1111/jvh.12766, PMID: 28772340

[29] Hammerich L Tacke F. Hepatic inflammatory responses in liver fibrosis. Nat Rev Gastroenterol Hepatol. (2023) 20:633–46. doi: 10.1038/s41575-023-00807-x 37400694

[30] Sanyal AJ Castera L Wong VW. Noninvasive assessment of liver fibrosis in NAFLD. Clin Gastroenterol Hepatol. (2023) 21:2026–39. doi: 10.1016/j.cgh.2023.03.042 37062495

[31] Ooi GJ Mgaieth S Eslick GD Burton PR Kemp WW Roberts SK. Systematic review and meta-analysis: non-invasive detection of non-alcoholic fatty liver disease related fibrosis in the obese. Obes Rev. (2018) 19:281–94. doi: 10.1111/obr.12628, PMID: 29119725

[32] Dulai PS Singh S Patel J Soni M Prokop LJ Younossi Z. Increased risk of mortality by fibrosis stage in nonalcoholic fatty liver disease: systematic review and meta-analysis. Hepatology. (2017) 65:1557–65. doi: 10.1002/hep.29085 28130788 PMC5397356

[33] Archer AJ Belfield KJ Orr JG Gordon FH Abeysekera KW. EASL clinical practice guidelines: non-invasive liver tests for evaluation of liver disease severity and prognosis. Front Gastroenterol. (2022) 13:436–9. doi: 10.1136/flgastro-2021-102064 PMC9380759 36051951

[34] Ciardullo S Muraca E Zerbini F Perseghin G. Liver stiffness is associated with all-cause mortality in patients with NAFLD: a systematic review and meta-analysis. Liver Int. (2023) 43:2604–10. doi: 10.1111/liv.15742 37724792

[35] Yoshioka K Hashimoto S Kawabe N. Measurement of liver stiffness as a non-invasive method for diagnosis of non-alcoholic fatty liver disease. Hepatol Res. (2015) 45:142–51. doi: 10.1111/hepr.12388 25040931

[36] Han S Choi M Lee B Lee HW Kang SH Cho Y. Accuracy of noninvasive scoring Systems in Assessing Liver Fibrosis in patients with nonalcoholic fatty liver disease: a systematic review and Meta-analysis. Gut Liver. (2022) 16:952–63. doi: 10.5009/gnl210391, PMID: 35193993 PMC9668505

[37] Ismaiel A Leucuta DC Popa SL Fagoonee S Pellicano R Abenavoli L. Noninvasive biomarkers in predicting nonalcoholic steatohepatitis and assessing liver fibrosis: systematic review and meta-analysis. Panminerva Med. (2021) 63:508–18. doi: 10.23736/S0031-0808.20.04171-3, PMID: 33165307

[38] Yun JW Cho YK Park JH Kim HJ Park DI Sohn CI. Hepatic steatosis and fibrosis in young men with treatment-naïve chronic hepatitis B. Liver Int. (2009) 29:878–83. doi: 10.1111/j.1478-3231.2009.01976.x, PMID: 19192167

[39] Yilmaz B Koklu S Buyukbayram H Yalçin K Korkmaz U Posul E. Chronic hepatitis B associated with hepatic steatosis, insulin resistance, necroinflammation and fibrosis. Afr Health Sci. (2015) 15:714–8. doi: 10.4314/ahs.v15i3.3, PMID: 26957957 PMC4765474

[40] Zheng RD Xu CR Jiang L Dou AX Zhou K Lu LG. Predictors of hepatic steatosis in HBeAg-negative chronic hepatitis B patients and their diagnostic values in hepatic fibrosis. Int J Med Sci. (2010) 7:272–7. doi: 10.7150/ijms.7.272 20714438 PMC2920573

[41] Huang Y Gan Q Lai R Wang W Guo S Sheng Z. Application of fatty liver inhibition of progression algorithm and steatosis, activity, and fibrosis score to assess the impact of non-alcoholic fatty liver on untreated chronic hepatitis B patients. Front Cell Infect Microbiol. (2021) 11:733348. doi: 10.3389/fcimb.2021.733348 35111690 PMC8801606

